# SENSORY DIFFICULTIES AND SOCIAL ISOLATION AMONG MEXICAN, US HISPANIC AND, US NON-HISPANIC INDIVIDUALS

**DOI:** 10.1093/geroni/igae098.2349

**Published:** 2024-12-31

**Authors:** Corinna Trujillo Tanner, Jeremy Yorgason, Rebekah Case Fankhouser, Jeana Olmo, Jase Wanlass, Kyriakos Markides, Joshua Ehrlich, Markus Wettstein

**Affiliations:** Brigham Young University, Provo, Utah, United States; Brigham Young University, Provo, Utah, United States; Brigham Young University, Provo, Utah, United States; Brigham Young University, Provo, Utah, United States; Brigham Young University, Provo, Utah, United States; The University of Texas Medical Branch, Galveston, Texas, United States; University of Michigan, Ann Arbor, Michigan, United States; Humboldt University Berlin, Berlin, Berlin, Germany

## Abstract

The Hispanic cultural value of Familismo describes strong identification and attachment of individuals with their families and is protective against social isolation. Hispanics comprise the largest minority group in the US and are more likely to live in intergenerational households. Sensory difficulty, including vision and hearing difficulty, are prevalent among older adults, disproportionately affecting Hispanic individuals. Research shows that aging and sensory difficulty are associated with an increased risk of social isolation. Using data from adults age 50 to 100 that participated in the Mexican Health and Aging Study, and Hispanic and non-Hispanic participants of the Health and Retirement Study, we explored how sensory difficulty related to social isolation. Social isolation was measured by assessing whether or not participants lived alone, had weekly contact with children and friends/relatives, and if they participated in weekly social activities. Findings suggested that vision and hearing difficulty rates were much higher among Mexican and US Hispanic individuals compared to US non-Hispanics. In contrast, social isolation was higher among US non-Hispanic individuals. Regression models with sensory difficulty predicting social isolation by country/ethnicity indicated that vision difficulty was associated with social isolation for Mexican and US non-Hispanic individuals, but not for US Hispanics. Hearing difficulty was associated with social isolation only for US non-Hispanic individuals. Among Mexican and US Hispanic individuals, it appears that age accounts for social isolation more so than sensory difficulty, and Familismo appears to protect against isolation. Among US non-Hispanics, sensory difficulty contribute to social isolation to a greater extent.

